# Elucidating molecular mechanisms of allergic sensitization to olive pollen through transcriptomics

**DOI:** 10.3389/fimmu.2026.1735045

**Published:** 2026-09-04

**Authors:** Pedro Chacón, Leticia Domínguez-Cereijo, Bouchra Doukkali, Lourdes Fernández-Delgado, Beatriz María Pérez-Machuca, Rosa Gil-Calderón, Adrián López-Postigo, Eloísa Andújar Pulido, Mónica Pérez Alegre, James R. Perkins, José Antonio Cornejo-García, Rajaa El Bekay, Nabil Hajji, Javier Monteseirín, Antonio Vega-Rioja

**Affiliations:** 1 Laboratorio de Inmunología y Alergia-FISEVI, UGC de Alergología. Hospital Universitario Virgen Macarena, Sevilla, Spain; 2 Departamento de Medicina, Facultad de Medicina, Universidad de Sevilla, Sevilla, Spain; 3 Genomic Unit, Andalusian Molecular Biology and Regenerative Medicine Center (CABIMER), CSIC, University of Seville, University Pablo de Olavide, Seville, Spain; 4 Departamento de Biología Molecular y Bioquímica, Facultad de Ciencias, Universidad de Málaga, Málaga, Spain; 5 Instituto de Investigación Biomédica de Málaga y Plataforma en Nanomedicina-IBIMA Plataforma BIONAND, Málaga, Spain; 6 Precision Allergy Translational Hub (PATH). Allergy Group, IBIMA Plataforma BIONAND, Málaga, Spain; 7 Allergy Unit, Malaga Regional University Hospital, Madrid, Spain; 8 Clinical Unit of Endocrinology and Nutrition, University Regional Hospital of Málaga, Málaga, Spain, , London, United Kingdom; 9 Spanish Biomedical Research Center in Physiopathology of Obesity and Nutrition (CIBERObn), Instituto de Salud Carlos III, Madrid, Spain; 10 Faculty of Medicine, Department of Surgery & Cancer, Imperial College London, London, United Kingdom; 11 Departamento de Bioquímica, Facultad de Medicina, Universidad de Sevilla, Sevilla, Spain; 12 Hospital Quirón Sagrado Corazón and Hospital Quirón Infanta-Luisa, Sevilla, Spain

**Keywords:** allergy, biomarkers, leukocytes, omics, therapeutic targets, transcriptomics

## Abstract

**Background:**

Olive pollen allergy, a leading cause of seasonal allergic rhinitis and asthma in Mediterranean regions, is becoming increasingly relevant worldwide due to the expansion of olive cultivation. Despite its significant public health impact, the molecular mechanisms underlying sensitization to olive pollen remain poorly understood.

**Methods:**

In this study, RNA sequencing was used to perform transcriptomic profiling on *in vitro-*stimulated Peripheral Blood Leukocytes (PBLs) from both allergic patients and healthy controls, to identify gene expression changes triggered by olive pollen in allergic individuals. Validation by RT-qPCR was subsequently performed in an independent cohort to confirm the expression patterns of selected DEGs.

**Results:**

A total of 37 differentially expressed genes (DEGs) were found exclusively in the patient group. Validation by RT-qPCR in an independent cohort confirmed the expression patterns of selected DEGs. Functional enrichment analyses associated these DEGs with immune pathways, ossification processes, and cytokine activity. Notably, upregulated genes such as *AREG*, *IL31RA*, and *IL18R1* were associated with TH_2_ responses and asthma pathogenesis, while downregulated genes including *GREM1* and *CCL1* were related to chemotaxis and immune regulation. Protein-protein interaction (PPI) network and hub gene analyses highlighted potential molecular drivers of allergic inflammation. Chromosomal mapping revealed clustering of DEGs on chromosomes previously associated with asthma susceptibility. Furthermore, exploratory correlation analyses identified correlation between TGM2 expression and total IgE, and between SHROOM2 expression and Ole e 7-specific IgE, highlighting these genes as candidates for future evaluation.

**Conclusion:**

Overall, this study provides new insights into the transcriptomic landscape of olive pollen allergy, identifying candidate biomarkers and biological pathways with translational relevance for improving diagnosis and advancing personalized treatment strategies in allergic diseases.

## Introduction

IgE-mediated allergic diseases pose a significant global health challenge due to their high prevalence, rising incidence, substantial impact on patients’ quality of life and socioeconomic costs (1). These diseases affect multiple organs and manifest in a wide range of clinical forms, from food allergies to respiratory disorders such as rhinitis and asthma. According to the World Health Organization (WHO), approximately 500 million people worldwide suffer from rhinitis, while nearly 300 million have asthma (2).

Pollen grains are among the most common triggers of respiratory allergies, with olive tree (*Olea europaea*) pollen being one of the main allergenic sources in the Mediterranean basin (3). In Spain, it is the second leading cause of pollinosis, with approximately 60% of pollen-allergic patients sensitized to olive pollen. In Andalusia, southern Spain, where olive cultivation is extensive, olive pollen allergy is the most prevalent respiratory allergy, affecting 84% of pollen-allergic individuals (4). During the pollination season (mid-April to late June), olive pollen concentrations can reach ext reme levels, triggering an increase in allergic respiratory disorders and pollinosis (5).

Olive tree pollinosis is increasing beyond the Mediterranean region due to the global expansion of olive cultivation and the growing popularity of the Mediterranean diet, which prominently features olive oil (6). Consequently, olive pollen has become a major allergenic source in California (U.S.), China, India, Australia, and South America (7), with the number of affected individuals increasing worldwide (6).

To date, 14 allergenic proteins have been identified in olive pollen, sequentially named Ole e 1 through Ole e 12, along with Ole e 14 (3, 8) and the recently described Ole e 15 (9). Ole e 1, the major allergen, is recognized by nearly 80% of individuals allergic to olive pollen (4). It is a glycoprotein that shares high similarity with other major allergens in the Oleaceae family (including *Fraxinus* and *Syringa* species) and it serves as a diagnostic marker for sensitization to this pollen family (10).

Beside Ole e 1, the prevalence of sensitization to olive Ags varies by geographic region and exposure levels (11). In regions with exceptionally high Ag exposure, olive pollen-allergic patients often show elevated specific IgE levels not only to Ole e 1 but also to minor allergens like Ole e 7 (a non-specific lipid transfer protein, nsLTP) and Ole e 9 (a glucanase), both linked to more severe asthma cases (12). Additionally, patients sensitized to Ole e 7 may develop pollen-food allergy syndrome due to cross-reactivity with nsLTPs found in Rosaceae fruits (13). In contrast, Ole e 9 sensitization has been associated with atopic dermatitis symptoms (14).

The transition toward personalized medicine highlights the crucial role of biomarkers in clinical practice, offering reliable, objective, and quantifiable indicators of disease presence, severity, and treatment response (15). While patient history, physical exams, and allergen-specific IgE testing remain the gold standard for diagnosing allergic diseases, there is a growing need for improved tools to support prediction, diagnosis, and monitoring. Although initially applied to cancer and monogenic disorders, omics technologies are now increasingly employed to identify biomarkers for allergic diseases (16). Genomics research, particularly genome-wide association studies (GWAS), has identified over 30 genetic loci associated with asthma susceptibility (17, 18). In contrast, transcriptomics provides a dynamic perspective of the complex heterogeneity of allergic diseases. Given that RNA expression varies with pathological states, transcriptomic analyses have identified distinct disease endotypes associated with severity, exacerbation risk, and responsiveness to therapies (19–22).

Although multi-omics strategies are being used increasingly in allergy research, to date only one study has analyzed the gene expression landscape in peripheral blood mononuclear cells of olive pollen–allergic patients across seasons (23), using microarrays. In this study, we used RNA sequencing (RNA-seq) to characterize the gene expression profile of *in vitro*–stimulated Peripheral Blood Leukocytes (PBLs) (lymphocytes, neutrophils, monocytes, eosinophils, and basophils) from patients sensitized to olive pollen. To minimize non-specific environmental exposure to olive and cross-reactive pollens, samples were collected outside the pollen season. We identified differentially expressed genes (DEGs), characterized their functional enrichment, and investigated their relationship with specific IgE levels to olive pollen and its allergenic proteins.

Our findings shed new light on the molecular mechanisms driving olive pollen allergy and highlight potential biomarkers for improved diagnosis and personalized management.

## Materials and methods

### Chemicals and reagents

Commercial lyophilized allergens including T_9_ (whole extract of *Olea europaea* pollen) were purchased from Roxall (Bilbao, Spain). Dextran T-500 and phosphate-buffered saline (PBS) were from Sigma-Aldrich Co. (Madrid, Spain). RPMI 1640 medium, fetal bovine serum, penicillin/streptomycin, amphotericin B, and N2 supplement were purchased from Thermo-Fisher (San Diego, CA, USA). All culture reagents had endotoxin levels of < 0.01 ng/ml, as tested by the Limulus lysate assay (Coatest, Chromogenix, Mölndal, Sweden).

### Patient and control selection

The study included two cohorts of donors: cohort 1 consisted of a group of atopic patients and healthy non-atopic controls (**Table 1**); and cohort 2 constituted of a group of atopic patients (**Supplementary Table 1**), recruited in the Allergy Unit of the Virgen Macarena University Hospital (Sevilla, Spain). All atopic patients were selected based on clinical history symptoms compatible with rhinitis, rhino-conjunctivitis and/or asthma to olive pollen. Diagnostic evaluation included a review of patient history, skin-prick test (SPT, Roxall), total IgE (ADVIA Centaur, Siemens, Germany) and serum specific-IgE (sIgE) (ImmunoCAP system, Thermo-Fisher). Patients were classified based on their specific antigen sensitization profile following sIgE measurement to the molecular components of olive pollen (Ole e 1, Ole e 7 and Ole e 9). Subjects had not undergone allergen immunotherapy and were instructed to avoid bronchodilators for at least 8 hours before *in vitro* assays (24 hours for oral bronchodilators). They had not used antihistamines, corticosteroids, disodium cromoglycate, or nedocromil sodium in the previous week. Additionally, they had not experienced asthma episodes for at least 3 months and had no respiratory infections in the 4 weeks prior to blood sampling. The healthy group had no history of allergies or bronchial symptoms, tested negative in skin prick tests, and had no serum-specific IgE to common inhalant allergens (house dust mites, pollens, molds, or animal dander). The study was approved by the Hospital Universitario Virgen Macarena Ethics Committee and adhered to the tenets of good clinical practice and principles of the Declaration of Helsinki. Written informed consent was obtained from each participant. All data were stored in an anonymized, coded database.

**Table 1:** Clinical and demographic characteristics of healthy donors (HD) and allergic patients (AP) in the initial cohort. .

Clinical and demographic characteristics	HD	AP
n	16	16
Age (max/min)*	41.25 ± 8.72 (63–25)	29.37 ± 13.96 (62–11)
Sex (male/female)	(4/12)	(8/8)
European ancestry	100%	100%
Total IgE (IU/ml)*	32.42 ± 12.86	605.01 ± 153.82
Positive skin tests	0%	100%
Olive-tree specific IgE (IU/ml)*	0.00 ± 0.00	55.17 ± 8.81
Ole e 1 specific IgE (IU/ml)*	0.00 ± 0.00	48.17 ± 10.22
Ole e 7 specific IgE (IU/ml)*	0.00 ± 0.00	18.24 ± 8.34
Ole e 9 specific IgE (IU/ml)*	0.00 ± 0.00	14.40 ± 6.72

*Mean ± SEM .

### Human leukocyte isolation and culture

Human PBLs were isolated from EDTA containing-tubes as previously described (24). Briefly, immediately after extraction, blood was mixed with Dextran T-500 (1.5%, w/v final concentration), which allows the sedimentation of most of the erythrocytes. The leukocyte-rich top layer was centrifuged and residual erythrocytes further removed by hypotonic lysis. Obtained cells were washed with PBS and cultured at 37 °C, in an atmosphere of 95% O_2_ and 5% CO_2,_ in complete medium (RPMI 1640 medium supplemented with N2 supplement, 2 mM L-glutamine, 100 U/ml penicillin, 100 µg/ml streptomycin and 250 ng/mL amphotericin B). For the treatments, PBLs (10^7^cells/ml) were incubated in the presence or absence of 10 µg/ml T_9_, for 18h. None of the reagents affected the viability of the cells at the concentrations used in this work, as confirmed by the trypan blue dye-exclusion test.

### RNA extraction

RNA samples were extracted from human PBLs using Trizol reagent (Invitrogen Thermo-fisher, CA, USA) and further purified using the RNeasy micro kit (Qiagen, CA, USA). RNA was quantified with a NanoDrop spectrophotometer (Thermo-Fisher) and NanoDrop IVD-1000 v.3.1.2 software (NanoDrop Technologies, Wilmington, DE, USA).

### RNA sequencing analysis and bioinformatics data analysis

RNA sequencing was performed by the Genomics Core Facility of CABIMER (Sevilla, Spain). The concentration and Integrity of RNAs were assessed with Qubit™ RNA HS assay (Thermo Fisher Scientific) and a 2100 Bioanalyzer System (Agilent, Santa Clara, CA, USA), respectively. Only samples with RNA integrity number ≥ 8 were selected.

The Total RNA-Seq libraries were generated using the Stranded Total RNA Prep Ribo Zero Plus kit (Illumina, Thermo-Fisher) according to the manufacturer’s instructions starting with 150 ng RNA. The sequencing was performed using the NovaSeq 6000 platform with 2 X 100 bp paired-end reads at a depth of 50 million reads per sample.

llumina Basespace Sequence Hub applications were used for data processing.

Demultiplexing, adapter trimming and conversion from BCL to FASTQ files were conducted using the FASTQ Generation app (bcl2fastq). Reads were mapped to the human genome (hg19) using the RNA-Seq Alignment app (v2.0.1) and differential gene expression analysis, comparing stimulated and unstimulated cells from patients and controls separately, was performed using DESeq2, implemented in the RNA-Seq Differential Expression App (v1.0.1). Differentially expressed genes (DEGs) were identified using a cut-off of adjusted *p*-value (false discovery rate) <0.1 and an absolute log_2_ fold change (log_2_FC ≥1 or log_2_FC) ≤-1. To understand the functional significance of the DEGs, gene enrichment analysis was performed on all upregulated and downregulated genes combined to capture globally affected biological pathways. The background gene set for the hypergeometric test was restricted to the custom transcriptomic background, consisting of all genes actively expressed and detected with non-zero counts across our peripheral blood leukocyte dataset. Gene sets were processed using ExpHunterSuite (25), together with specific databases (Gene Ontology, Kyoto Encyclopedia of Genes and Genomes-KEGG, Reactome, and DisGeNET (26). The Search Tool for the Retrieval of Interacting Genes/Proteins, STRING online tool (https://string-db.org) was used to construct the PPI network, and Cytoscape software (https://cytoscape.org) was employed to visualize the interaction results from STRING. To identify hub genes, the CytoHubba plugin was applied using the degree method. The relationships between chromosome locations, gene biotypes, and expression levels of the DEGs were visualized using the circlize package in R (27). The circular plot is composed of concentric tracks arranged from the outside to the inside. The outermost track (Track 1) displays gene labels positioned according to their genomic locations, followed by Track 2 showing their corresponding chromosomes. Track 3 presents the chromosome ideogram, while the innermost track (Track 4) visualizes the fold change values associated with each gene.

**Figure 1:**
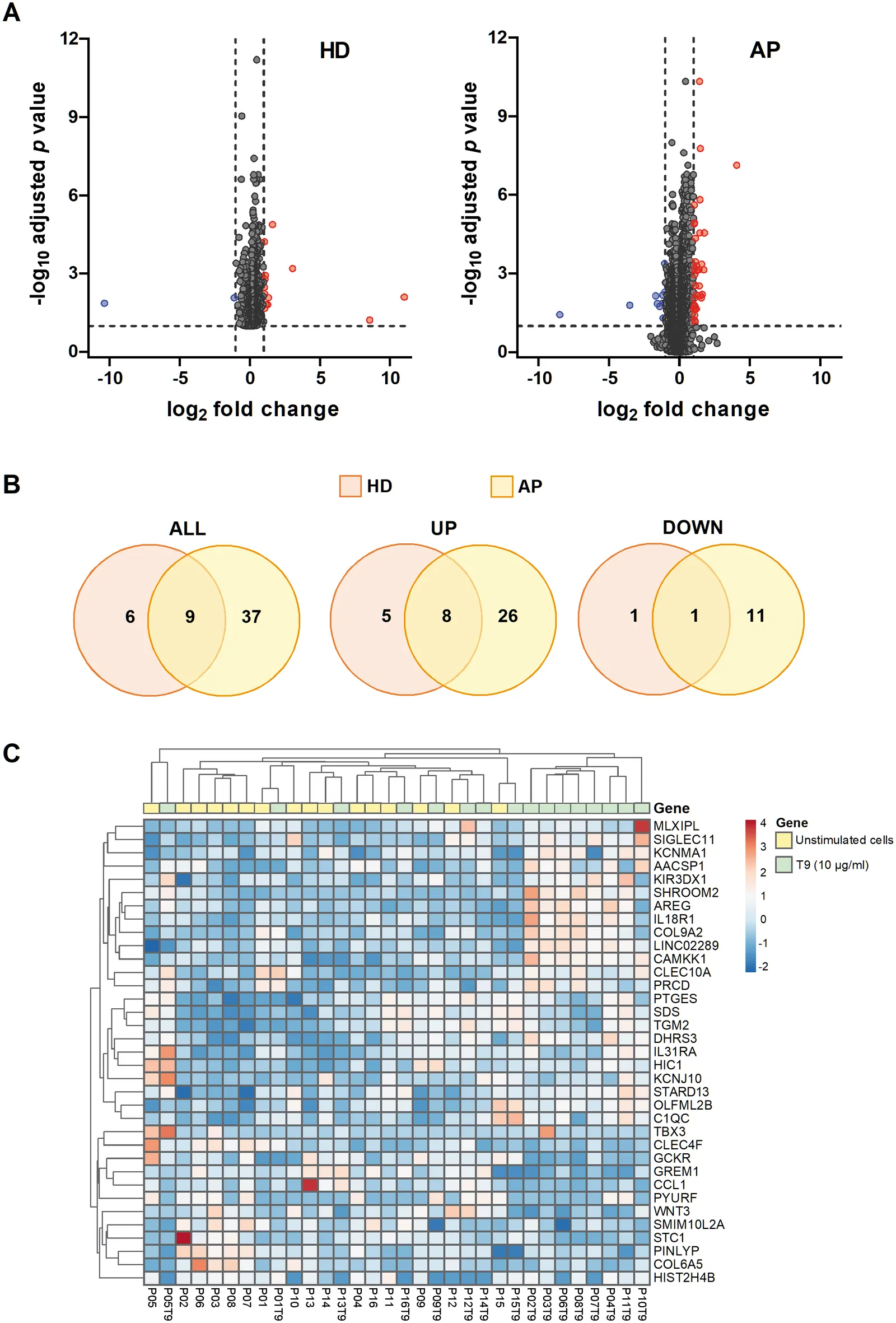
Identification of differentially expressed genes (DEGs). **(A)** Volcano plots for healthy donors (HD, left) and allergic patients (AP, right). Red dots indicate upregulated genes, blue dots indicate downregulated genes, and gray dots indicate genes with no significant expression changes. **(B)** Venn diagrams illustrating genes unique to or shared between HD and AP. Left: all DEGs (ALL); middle: upregulated DEGs (UP); right: downregulated DEGs (DOWN). **(C)** Expression patterns of the 37 AP-exclusive DEGs. Patients are shown in columns before (yellow) and after T_9_ treatment (green), while DEGs are displayed in rows. Red and blue colors represent upregulation and downregulation, respectively. The heatmap was generated using ClustVis 2.0 (http://biit.cs.ut.ee/clustvis/). Hierarchical clustering was performed using ClustVis 2.0 based on Euclidean distance and the average linkage method. The color scale displays row-scaled Z-scores calculated from normalized RNA-seq counts, where red indicates relative upregulation and blue indicates relative downregulation.

For expression profile visualization, hierarchical clustering for **Figure 1C** was performed using ClustVis 2.0 based on Euclidean distance and the average linkage method. The corresponding color scale represents row-scaled Z-scores calculated from normalized RNA-seq counts, where red indicates relative upregulation and blue indicates relative downregulation.

### Retrotranscription and quantitative PCR

1 µg total RNA was reverse transcribed into cDNA using random hexamers (Promega, WI, USA) and the Superscript III first-strand synthesis system (Thermo-Fisher). qPCR was performed using the StepOne system (Applied Biosystems, Thermo-Fisher). Human gene expression analysis was performed using specific TaqMan probes and the housekeeping gene rRNA18S (Applied Biosystems, **Supplementary Table 2**). Relative gene expression levels were calculated using the 2^-ΔΔCt^ method and the housekeeping gene for normalization as previously described (28).

### Statistical analysis

The minimum RNA-Seq sample size was estimated based on the method described by Hart et al. (29). This approach determined that a sample size of n = 9 per group is required to detect a 2-fold change in gene expression (δ=2) with a significance level of α = 0.05 and a statistical power of 90% (β=0.1). The calculation assumed a sequencing depth of 50 million reads per sample (μ=50) and a coefficient of variation (σ) of 0.43, representing the average biological variation in humans, as estimated by Hart et al., based on 12 human RNA−Seq datasets. Based on this minimum requirement, we selected a sample size of 16 patients and 16 healthy controls for our study to achieve a statistical power of 99%. Differential expression analysis of RNA-seq data was performed as described above. All further statistical analyses were performed using GraphPad Prism 8.0.1 (GraphPad Software, San Diego, CA, USA). The Shapiro–Wilk test was used to assess the normality of variable distributions. Depending on data distribution, either Student’s t test (parametric) or the Mann-Whitney U test (non-parametric) was used for group comparisons. Data are expressed as means ± SEM. A level of p<0.05 was considered significant. *: p<0.05; **: p<0.01; ***: p<0.001. Associations between gene expression and total IgE, as well as IgE specific to olive pollen and its molecular components, were evaluated using Spearman rank correlation coefficients. P-values were adjusted for multiple testing using the Benjamini–Hochberg false discovery rate (FDR) procedure. For visualization purposes, gene-IgE pairs with a correlation coefficient **ρ** > 0.5 were selected and displayed graphically. For these correlation analyses, gene expression changes were computed as the log__2__ ratio of RNA-seq counts from antigen-stimulated cells to those from unstimulated cells from the same allergic patient.

## Results

### Identification of olive pollen-responsive genes

To explore the molecular mechanisms of olive pollen allergy, RNA sequencing was performed on blood PBLs from an initial cohort of allergic patients and healthy controls (See **Table 1** for demographic and clinical details). Transcriptional profiles were analyzed after 18 h of *in vitro* culture with or without 10 µg/ml T_9_ in both groups.

Differentially expressed genes (DEGs) were identified using an adjusted *p*-value <0.1 and an absolute fold change of ≥2 (log_2_FC) ≥1 or log_2_FC) ≤-1). A total of 46 DEGs were detected in allergic patients, while 15 were found in healthy donors. Volcano plots (**Figure 1A**) showed that in allergic patients, 34 of the 46 T_9_- differentially expressed genes were upregulated, while 12 were downregulated. In healthy donors, 13 of the 15 T_9_-regulated genes were upregulated, and 2 were downregulated. Full DEG lists for allergic patients, and healthy donors are provided in **Supplementary Table 3** and **4**, respectively.

Interestingly, as shown in Venn diagrams (**Figure 1B**), most T_9_-regulated genes (37 of 46) were exclusive to allergic patients.

To assess the consistency of patients’ transcriptional responses to antigen stimulation, we performed hierarchical clustering using the 37 DEGs exclusively regulated in allergic patients, using paired stimulated (T_9_-treated) and unstimulated samples. As shown in **Figure 1C**, nine of the sixteen T_9_-treated samples formed a tight cluster, reflecting a homogeneous and robust response to stimulation. The remaining seven treated samples, by contrast, intermingled with their corresponding unstimulated controls, often clustering most closely with the untreated sample from the same patient. This highlights inter-individual variability in transcriptional activation, suggesting heterogeneity in immune activation profiles.

### Functional enrichment analysis of DEGs

**Figure 2:**
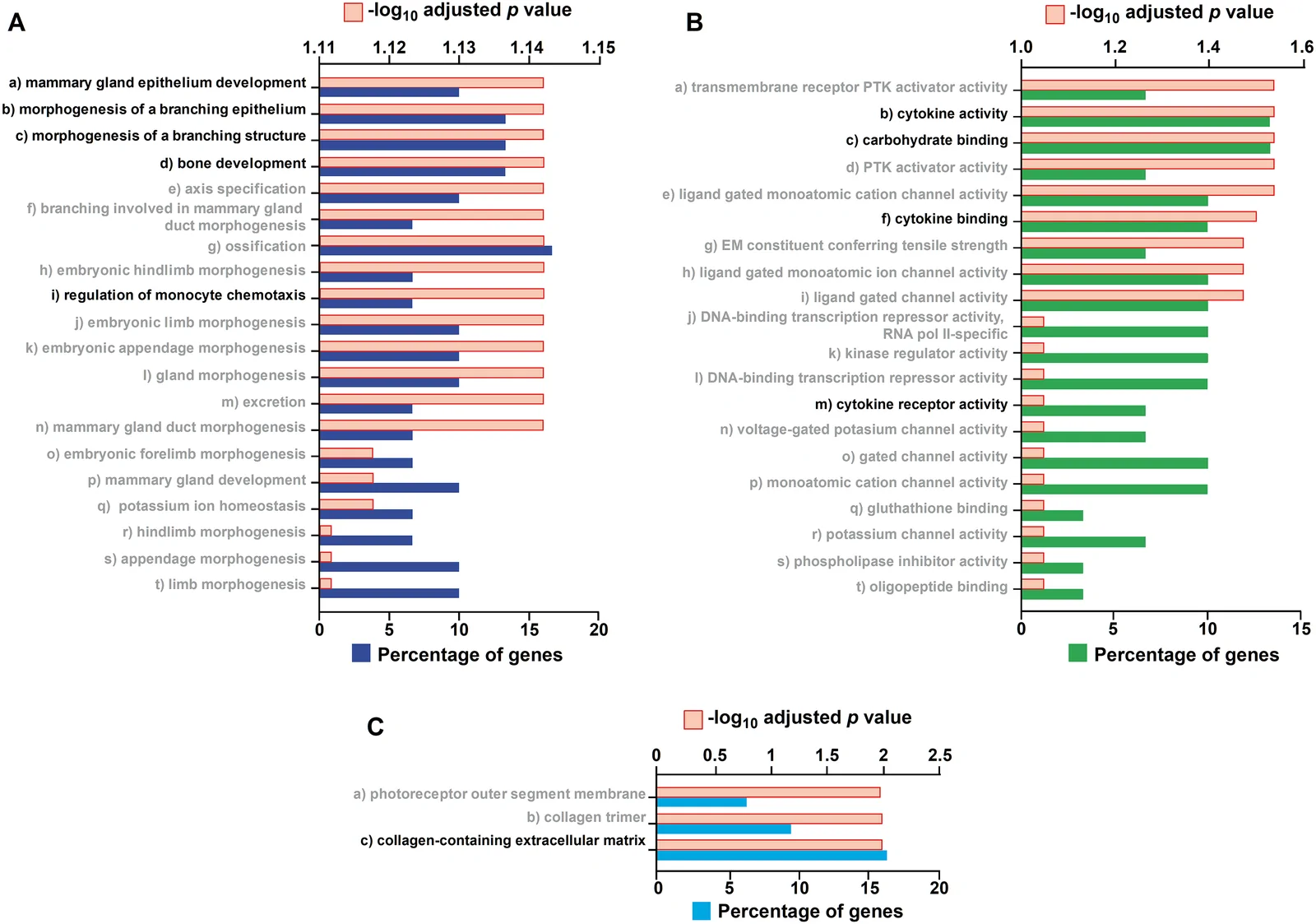
Gene ontology (GO) functional enrichment analysis. Top 20 terms of the biological process (BP) **(A)** and molecular function (MF) **(B)** categories. Terms of the cellular component (CC) **(C)** category. GO analysis associated with the olive pollen DEGs was performed using ExpHunterSuite. Red bar lines represent adjusted p-values for the top-ranked terms. They were calculated using hyper-geometric tests, corrected by Bonferroni adjustment and expressed as the negative logarithm (base 10). Dark blue **(A)**, green **(B)** or light blue **(C)** bar lines represent the percentage of gene within each term. Pathways in bold are discussed in the text.

To investigate the biological roles of the DEGs in response to olive pollen, we performed Gene Ontology (GO) enrichment analysis, across the three functional categories: biological process (BP), molecular function (MF), and cellular component (CC). Among the 46 DEGs, 31 were linked to significantly enriched GO terms. The top 20 terms for each category are shown in **Figure 2**.

For BP (**Figure 2A**), DEGs were mainly associated with ossification (GO:0001503), involving 16.66% of the genes (adjusted p-value: 7.0 ×10^-2^), followed by morphogenesis of a branching epithelium, morphogenesis of a branching structure, and bone development (GO:0061138, GO:0001763, GO:0060348), each comprising 13.33% of the genes (adjusted p-value: 7.0 ×10^-2^). The only immune-related BP was the regulation of monocyte chemotaxis (GO:0090025; 6.6% of the genes, adjusted p-value: 7.0 ×10^-2^), including downregulated genes such as *GREM1* and *CCL1*.

For MF (**Figure 2B**), the most enriched terms were carbohydrate binding (GO: 0030246; 13.3% of the genes, adjusted p-value: 2.8 ×10^-2^) and cytokine activity (GO: 0005125; 13.3% of the genes, adjusted p-value: 2.8 ×10^-2^). The cytokine activity category included the upregulated gene *AREG* and the downregulated genes *WNT3*, *GREM1*, and *CCL1*. Other related MF categories included cytokine binding (GO: 0019955; 10% of the genes, adjusted p-value: 3.1 ×10^-2^) and cytokine receptor activity (GO: 0004896; 6.6% of the genes, adjusted p-value: 8.9 ×10^-2^), which featured upregulated genes like *IL31RA*, *GREM1*, and *IL18R1*.

For CC (**Figure 2C**), only three terms were enriched, with the collagen-containing extracellular matrix (GO: 0062023) showing the highest representation (16.13% of genes, adjusted p-value: 1 ×10^-2^).

The links between the enriched GO terms and the associated genes across all three categories, BP, MF, and CC, are presented in **Supplementary Figure 1**. Considering the top nodes along with their associated genes, *GREM1* emerged as a downregulated gene shared between BP and CC, indicating its potential involvement in multiple biological functions.

In addition to the GO analysis, pathway analysis and the association of DEGs with human diseases were performed using KEGG, Reactome and DisGeNET respectively, but no significant results were obtained.

### Protein-protein interaction networks and circular visualization of DEGs

**Figure 3:**
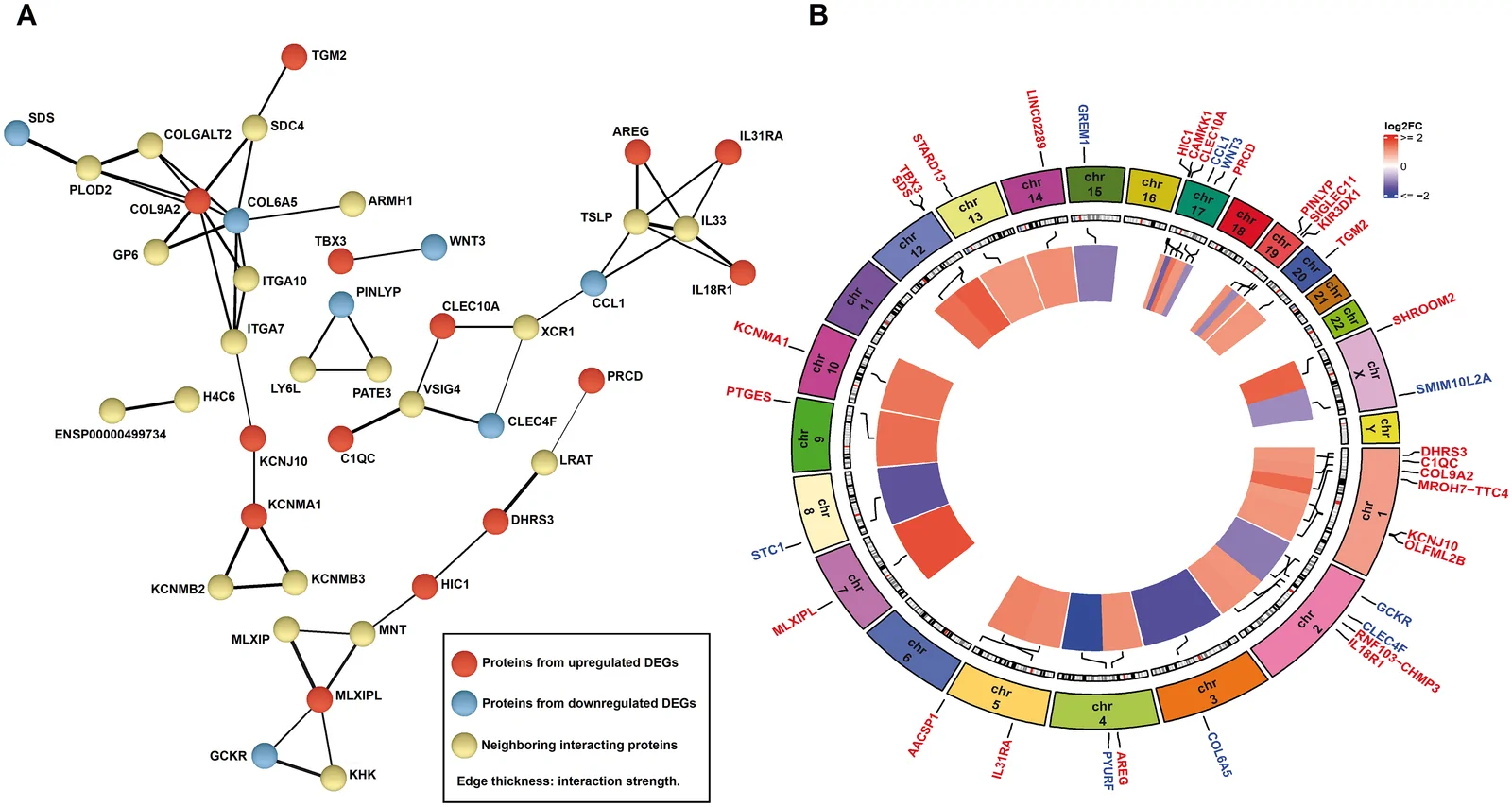
Protein-protein interaction (PPI) network analysis and chromosomal distribution of DEGs. **(A)** PPI network of all DEGs. Nodes represent proteins, while edges indicate functional and physical interactions, with edge thickness reflecting interaction strength. Red nodes correspond to proteins encoded by upregulated DEGs, blue nodes represent proteins from downregulated DEGs, and yellow nodes show 20 neighboring interacting proteins. **(B)** Circos plots illustrating the distribution of DEGs across human chromosomes. The outermost track (Track 1) displays gene labels positioned according to their genomic locations, followed by Track 2 showing their corresponding chromosomes. Track 3 presents the chromosome ideogram, while the innermost track (Track 4) visualizes the fold change values associated with each gene. Red/blue color intensity encodes the magnitude and direction of the log2 fold change (log2FC) values (red for upregulation, blue for downregulation).

To understand the interactions between the functional proteins encoded by the DEGs, PPI networks were identified using STRING. Given the limited number of DEGs identified in our results, interacting neighbour genes were included in the network construction. A minimum interaction score with an average confidence of 0.4 was applied, and the number of interactors was limited to a maximum of 20. The generated network contained 42 nodes and 53 edges, with a PPI enrichment p-value of 1.12 ×10^-5^ (**Figure 3A**). To further explore genes that may play an important role in allergy to olive tree pollen, the CytoHubba plugin was used to identify hub genes. The most important hub genes in the PPI network were identified using the degree-based method (**Supplementary Figure 2**).

In addition, Circos plots were utilized to visualize the global distribution and chromosomal localization of the identified DEGs across the human genome (**Figure 3B**). The directional trend and magnitude of expression changes are shown by the red/blue color intensity of the data points, where red signifies relative upregulation and blue represents relative downregulation. Based on this structural distribution, chromosomes 1, 2, 17, and 19 were found to contain the highest number of DEGs responsive to the allergen challenge.

### Gene expressions validation by RT-qPCR analysis

To validate the RNA-seq findings, we replicated the gene expression changes in an independent cohort of five olive pollen allergy patients with similar clinical characteristics to the initial group (**Supplementary Table 2**) using RT-qPCR analysis. Target genes were selected from the DEGs in the initial cohort based on fold changes, immune system relevance, GO term enrichment, and identification as hub genes. These included upregulated genes (*AREG*, *IL31RA*, *IL18R1*, *MLXIPL*, *KCNMA1*) and downregulated genes (*GREM1*, *CCL1*, *GCKR*). The analysis was extended to include additional genes previously associated with allergy and/or asthma, including upregulated genes (*SHROOM2*, *TGM2*) and downregulated genes (*STC1*, *CLEC4F*) (30–35). Notably, these genes were identified as differentially expressed genes (DEGs) in our transcriptomic analysis but were not captured in the Gene Ontology (GO) term enrichment results, likely due to enrichment thresholds and/or incomplete GO annotation. Nevertheless, they were retained based on their well-established relevance in allergy-related processes. While the results aligned with RNA-seq data, differences in fold changes were observed between the two methods. These variations were mainly due to the higher sensitivity of qPCR, which consistently produced larger fold changes than RNA-seq.

**Figure 4:**
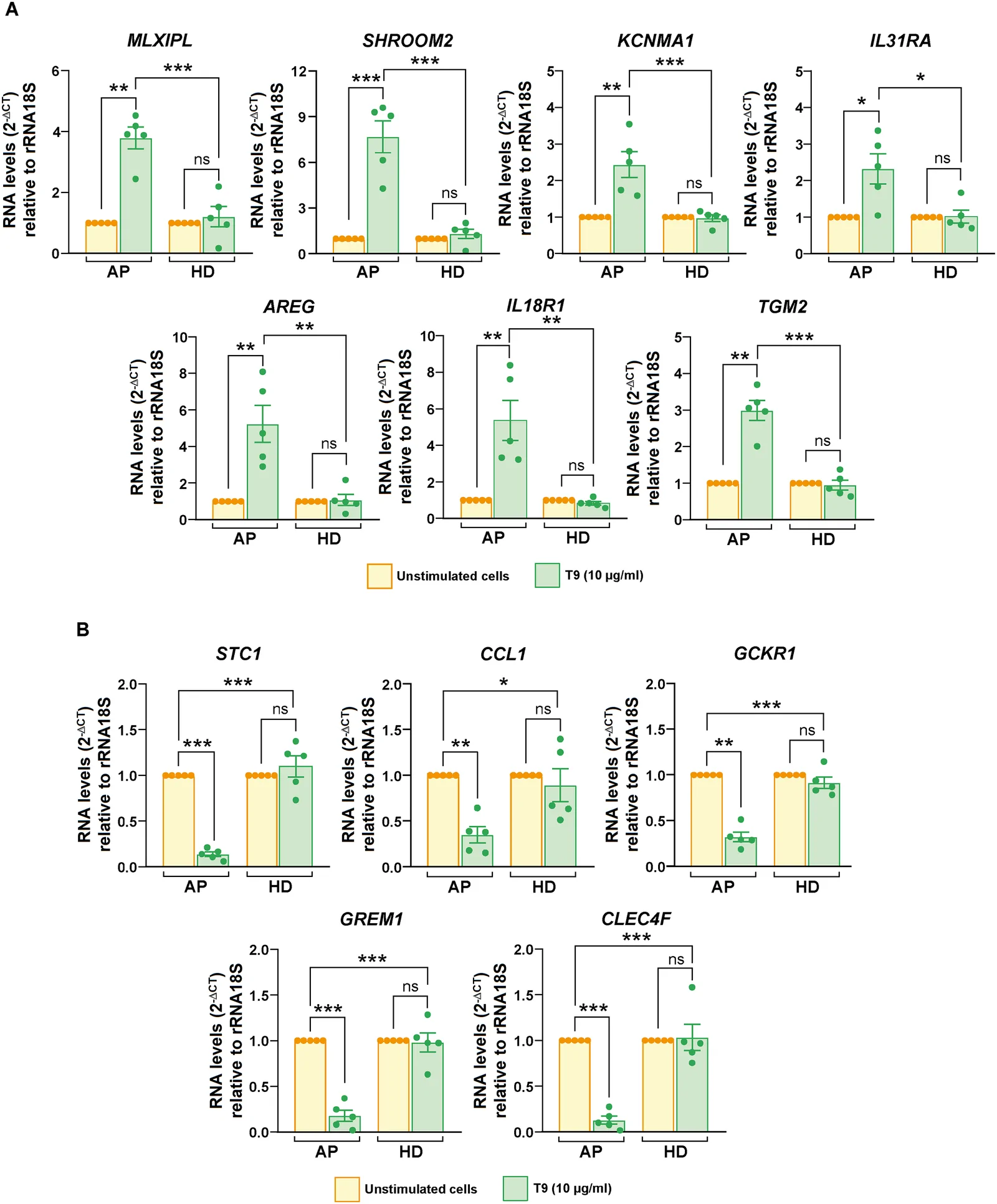
Validation of upregulated DEGs by RT-qPCR. The mRNA expression of selected upregulated **(A)** and downregulated **(B)** DEGs was assessed by rRT-qPCR in PBLs from olive-tree allergic patients (AP, n=6) and healthy donors (HD, n=6). Expression levels were normalized to 18S rRNA, which served as the housekeeping reference gene. The bar graph represents the average fold induction ± SEM from five independent experiments, each performed in triplicate. Statistical analysis was conducted using ordinary one-way ANOVA with multiple comparisons via *t*-tests without correction (Fisher’s LSD test). Brackets indicate the comparisons made. Statistical significance is denoted as follows: (*) p < 0.05, (**) p < 0.01, (***) p < 0.001.

As shown in **Figure 4**, all selected target genes evaluated for validation showed statistically significant expression changes after olive pollen exposure in the allergic patient group, whereas no significant differences were observed for these specific genes in healthy donors. Among the upregulated genes (**Figure 4A**) -all showing approximately ≥2-fold increase- *SHROOM2*, *IL18R1*, and *AREG* displayed the highest relative expression levels with increases of 7.67-, 5.36-, and 5.24-fold, respectively. For the downregulated genes (**Figure 4B**), all showed more than a 2-fold decrease (0.5-fold), with *CLEC4F*, *STC1*, and *GREM1* experiencing the greatest reductions (0.13, 0.12, and 0.17-fold decreases, respectively).

### Correlation of biomarker expression in olive pollen asthmatic patients

**Figure 5:**
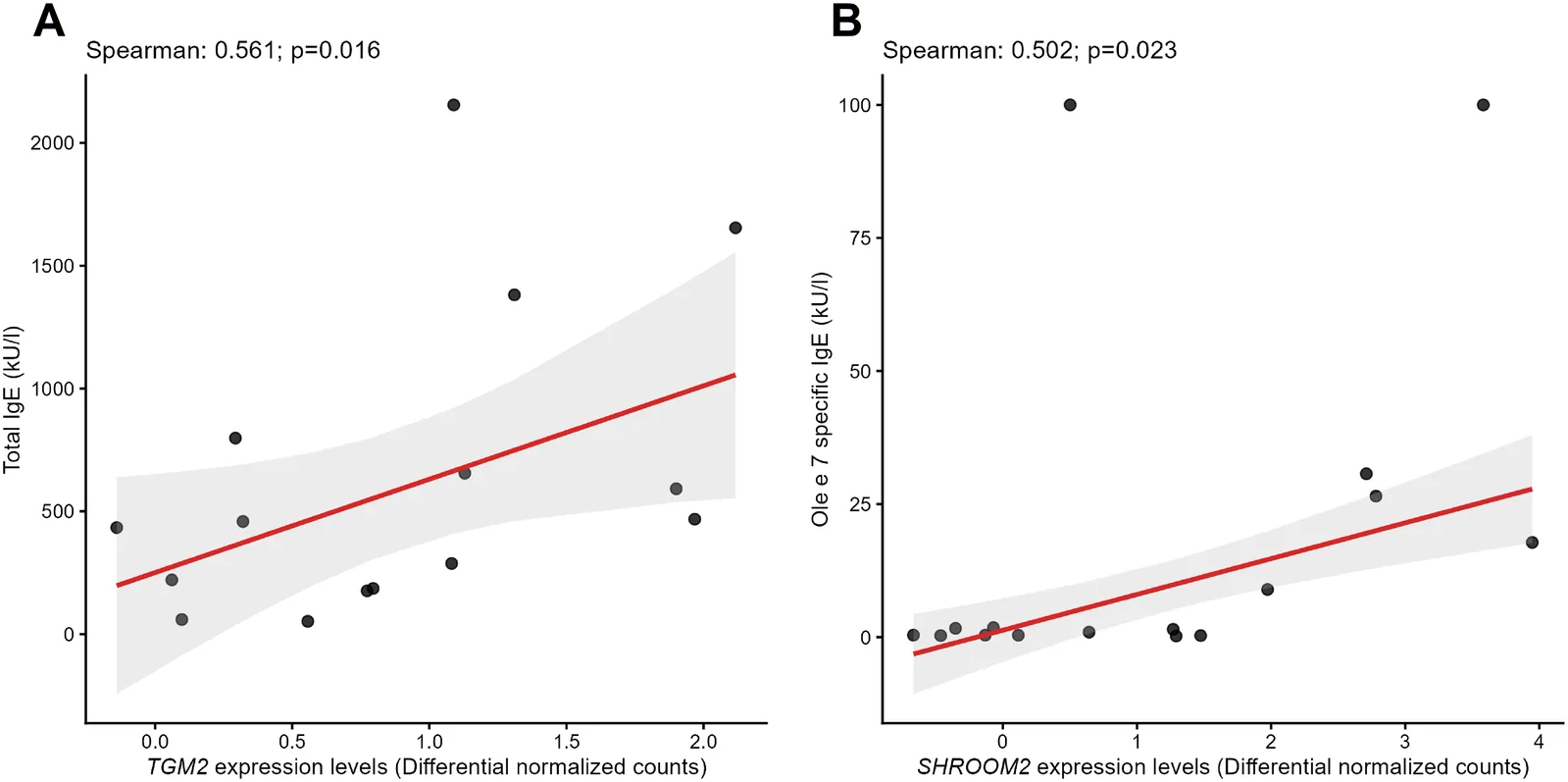
Correlation analysis between DEG expression and serum total **(A)** or specific **(B)** IgE levels in olive tree allergic patients. **(A)** Scatter plot depicting *TGM2* expression levels and serum total IgE levels in the initial cohort. **(B)** Scatter plot depicting *SHROOM2* expression levels and specific IgE levels in the initial cohort. As we employed Spearman correlation, we included a robust linear fit (RLM) with 95% confidence intervals for visualization purposes only. Correlation coefficients (r) and were calculated using Spearman’s rank correlation. Although the nominal p-values were < 0.05, they were not significant following multiple hypothesis correction.

To assess the potential of the validated target genes as biomarkers for olive pollen allergy, exploratory association analyses were performed between changes in their mRNA expression and total and allergen-specific IgE levels in allergic patients from the initial cohort. A positive correlation was observed between changes in *TGM2* expression and total serum IgE levels (Spearman’s **ρ** = 0.5607, nominal p = 0.016), but this association did not remain significant after multiple hypothesis correction. (**Figure 5A**). No correlation was found between gene change and specific IgE to the whole olive pollen extract.

Further analysis of specific IgE levels against individual molecular components of the extract revealed a nominal positive correlation between *SHROOM2* expression and Ole e 7-specific IgE (Spearman’s **ρ** = 0.5022, p = 0.023), which did not remain significant after multiple testing correction (**Figure 5B**).

## Discussion

The development of novel biomarkers is crucial for improving asthma diagnosis, classification, and treatment (36). Multiomics, particularly transcriptomics, has enabled the discovery of new biomarkers useful for distinguishing asthma phenotypes and predicting treatment responses (37, 38). For example, CST1 is a potential biomarker for airway allergic diseases, including asthma comorbid with allergic rhinitis (39). Integrating these biomarkers into clinical practice could enhance personalized treatment strategies.

Olive pollen is a major trigger of rhinitis and asthma in the Mediterranean and is becoming more prevalent globally (3, 7). While sIgE to Ole e 1, 7, and 9 aids in characterizing phenotypes and cross-reactivities, these markers have limited diagnostic accuracy and value in guiding AIT (40). To date, only one microarray-based study has explored gene expression in olive pollen allergy, focusing on peripheral blood mononuclear cells and excluding granulocytes (23). In our study, however, by analysing whole blood PBLs, we are able to capture a broader immune profile. In addition, in that study they analysed the expression changes during natural exposure and did not account for geographic pollen variation or co-exposure to overlapping allergens like Platanus and Poaceae, which may affect results in polysensitized individuals (41).

This work represents the first comprehensive transcriptomic analysis of allergic sensitization to olive pollen in *in vitro*-stimulated whole blood PBLs. We identified 37 DEGs uniquely changing in allergic individuals following olive pollen exposure, which were not observed in healthy controls, suggesting a sensitization-specific transcriptomic profile and the potential of transcriptomics to reveal disease-specific signatures (1, 11). In mixed primary clinical samples like whole PBLs, transcriptomic responses to *in vitro* allergen challenge are characteristically subtle and subject to high inter-individual variance compared to purified cell lines. Consequently, an adjusted p-value threshold of < 0.1 is standard and necessary to avoid Type II statistical errors. The robustness of our discovery pipeline is shown by our independent cohort, where 100% of the selected candidates achieved high statistical significance under RT-qPCR. Hierarchical clustering showed a consistent and shared transcriptional response in the majority of patients, while a subset of patients exhibited minimal changes, suggesting a similar expression profile before and after stimulation. This heterogeneity may be due to variations in disease severity, immune priming, allergen exposure history or genetic background, underscoring the need for personalized allergy management (42).

Unexpectedly, functional enrichment analysis of DEGs linked them to biological processes related to ossification, morphogenesis, and bone development. While systemic inflammation in chronic allergic diseases has been epidemiologically linked to bone remodeling disorders like osteoporosis (43), these short-term *in vitro* transcriptomic shifts in mature circulating leukocytes more likely reflect the activation of highly pleiotropic signaling cascades -such as the BMP/TGF-β pathways- which are heavily cross-utilized by immune cells for transdifferentiation, cytoskeletal reorganization, and cellular migration during acute allergen challenge. Immune-related GO terms, including monocyte chemotaxis regulation, were also enriched, highlighting immune cell recruitment in allergic responses. Downregulated genes like *GREM1* and *CCL1*, both involved in chemotaxis, may contribute to dysregulated immunity. *GREM1*, a BMP antagonist, regulates monocyte adhesion, migration, and survival (44). Although its role in allergy remains elusive, the closely related gene *GREM2* is downregulated in bronchial biopsies of asthma patients versus control (45). Likewise, *CCL1*, upregulated in mast cells after FcϵRI crosslinking, attracts CD4^+^ T cells and has been implicated in allergic inflammation (46).

Enriched MF identified the cytokine-related key molecules AREG, IL31RA, and IL18R1, pointing toward a broader involvement in immune signalling. AREG, (Amphiregulin), is a well-recognized TH_2_-associated cytokine biomarker. Elevated levels of AREG have been observed in dendritic cells from allergic individuals (47)), and it is implicated in the development and progression of asthma (48). IL31RA instead has a role in promoting airway hyperresponsiveness, through the smooth muscle contraction (49). IL18R1 has been associated with genetic polymorphisms related with asthma and bronchial hyperreactivity (50). Recent findings also link IL18R1 to severe and neutrophilic asthma phenotypes, highlighting its upregulation in these specific conditions (51, 52). The intrinsic biological robustness of these findings is further supported by network topology analysis. The centrality of *AREG*, *IL31RA*, and *IL18R1* as hub genes in the network suggests a potential coordinate role in managing the downstream immune signaling cascades triggered by pollen exposure.

Apart from the functional enrichment, these three genes also emerged as central hubs in the PPI network together with *KCNMA1*. This gene encodes for the α-subunit of the maxi-K channel and has been primarily involved in airway smooth muscle regulation. Dysfunction in *KCNMA1* has been linked to airway hyperreactivity, asthma severity, and rhinosinusitis (53–55). These findings suggest a complex interplay between cytokine signaling and ion channel activity in asthma pathogenesis.

Beyond the identified pathways, our analysis revealed novel candidate genes -*MLXIPL* and *GCKR*- potentially involved in allergic inflammation. This highlights a possible connection between metabolic regulation and immune responses in allergy and asthma. Both genes are central regulators of cellular energy metabolism, which regulates immune cells function in chronic inflammatory processes (56). *MLXIPL* encodes the carbohydrate response element-binding protein (ChREBP), a transcription factor that regulates glucose-induced gene expression. Recent evidence suggests that ChREBP not only promotes glycolytic flux but also facilitates immune cell migration through mTOR pathway activation (57). This is particularly relevant in the context of allergic inflammation, where metabolic reprogramming supports the bioenergetic demands of activated immune cells such as TH_2_ cells and dendritic cells. *GCKR*, which encodes the glucokinase regulatory protein (GKRP), plays a critical role modulating glucokinase activity and thereby controlling glucose utilization. Kishore et al. demonstrated that GKRP influences the migration of regulatory T cells (T_regs_) (58), though the control of glycolytic pathways. Given the immunoregulatory role of T_regs_ in allergic disease, the downregulation of *GCKR* may suggest a potential impairment in T_reg_-mediated immune suppression, thereby contributing to persistent inflammation.

In addition to the enriched and hub DEGs, we explored two others downregulated genes, *STC1* and *CLEC4F*, which offer insights into the regulation of asthma and immune responses. *STC1* (Stanniocalcin-1) is decreased in the serum of asthma patients, which correlates with poorer disease control and reduced lung function (34), suggesting a potential protective or regulatory role in respiratory inflammation. *CLEC4F*, a C-type lectin receptor predominantly expressed in non-classical monocytes (33), modulates TH_2_-mediated allergic inflammation, likely through GM-CSF induction (35).

To gain further insight into the genomic landscape of the DEGs, we conducted a chromosomal circular visualization analysis. This approach revealed clustering on chromosomes 1, 2, and 17-well known allergy and asthma-related regions. Chromosome 1 harbors *DENND1B* (1q31), a gene implicated in memory T-cell function and associated to asthma susceptibility (59). On chromosome 2, *IL1RL1* (2q11), which encodes the receptor for IL-33, plays a key role in TH_2_ cell activation and type 2 inflammation (60). Chromosome 17q21 stood out as the most enriched region, consistent as the most linked asthma locus. It contains *ORMDL3* and *GSDMB*, both involved in airway remodeling and inflammatory processes (61). Interestingly, our analysis also revealed DEGs clustering on chromosome 19, a region less commonly associated with allergic disease. This finding suggests that previously underexplored genomic loci may be involved in the regulation of allergic responses, which warrants further investigation.

Our study also explored the relationship between DEGs and clinical parameters, such as serum IgE levels. *TGM2* expression showed nominal correlation with total IgE, although it was not significant following multiple testing correction. TGM2 is a multifunctional enzyme that catalyzes Ca²^+^-dependent protein modification, and regulates immune signaling, apoptosis, and cell adhesion (62). Elevated in egg allergy (31) and linked to asthma phenotypes (30), *TGM2* has shown therapeutic promise, with inhibitors improving asthma in experimental models (63). However, its lack of correlation with olive-specific IgE suggests it is not pollen-specific. Unlike *TGM2*, *SHROOM2* expression showed a nominal correlation with specific IgE levels to the olive pollen nsLTP Ole e 7. However, these findings are preliminary and that further research is needed to assess the potential of *SHROOM2* as a specific biomarker for allergy to this olive pollen component and/or as a broader biomarker for other nsLTPs. *SHROOM2* encodes an actin-binding protein that regulates cell motility and cytoskeletal remodeling (64). Both processes are involved in immune cell function and airway dynamics. Actin cytoskeletal remodeling is implicated in airway constriction and inflammation in asthma. Thus, it is plausible that *SHROOM2* may influence allergic responses through similar mechanisms. Indeed, a single study showed an altered methylation of *SHROOM2* in animal models of asthma, hinting at a potential role in the epigenetic regulation of allergic inflammation (32). Although its precise function in respiratory allergy remains unclear, *SHROOM2* emerges as an intriguing candidate for further research into the cellular and epigenetic underpinnings of allergen-specific immune responses.

A potential limitation of this study is the age difference between the two cohorts (28.00 ± 4.18 *vs* 45.40 ± 6.08 years). However, this is unlikely to affect the findings, as evidence suggests that total serum IgE levels remain relatively stable throughout adulthood. Additionally, data processing and alignment natively utilized the hg19 human reference genome to maintain strict pipeline uniformity and backward compatibility with historical datasets in our core facility. A comprehensive lift-over cross-reference confirmed that all 37 unique DEGs map without any structural ambiguity or nomenclature discrepancy to current GRCh38 coordinates, ensuring that the assembly version did not introduce analytical bias. Previous studies have reported no consistent or clinically significant age-dependent effects on IgE production in young and middle-aged populations (65), indicating that age was not a major confounding factor in our results.

In conclusion, this study offers valuable insights into the immune response to olive pollen and highlights possible potential biomarkers that could enhance diagnosis and enable more personalized treatment approaches. By using an *in vitro* stimulation model, our study avoids seasonal exposure variability and allows for standardized allergen challenge conditions. Nonetheless, several limitations should be acknowledged. The sample size, while adequate for an initial discovery phase, requires validation in larger, multicenter cohorts. Furthermore, the use of peripheral blood-stimulated PBLs may not fully reflect tissue-specific immune responses, underscoring the need for future studies focusing on resident immune cells from induced sputum or bronchoalveolar lavage samples.

Further research is also needed to elucidate the roles of DEGs, particularly those associated with ossification, bone development, and metabolism, which may represent systemic effects of allergic inflammation. Mechanistic studies will be essential to determine their relevance to allergic sensitization. Additionally, the diagnostic, prognostic, and therapeutic potential of these DEGs in olive pollen allergy should be explored through longitudinal studies. Integrating transcriptomic data with proteomic and metabolomic analyses could provide a more comprehensive view of olive pollen allergy and facilitate the development of personalized therapeutic strategies.

## Data Availability

The raw RNA-Seq data generated in this study have been deposited in the European Nucleotide Archive (ENA) under the project accession number PRJEB122644.

## Supplementary material

Supplementary Figure 1 Network representations of the relationship among GO enriched terms. The network connects the top ranked functional terms for biological process (BP, dark blue nodes) **(A)**, molecular function (MF, green nodes) **(B)** and cellular component (CC, light blue nodes) **(C)** categories to their associated genes (grey nodes). The size of the functional term shows the number of connected genes.

Supplementary Figure 2 Identification of Hub Genes. Network of 30 hub genes identified using the Degree algorithm in the cytoHubba plugin. Red boxes indicate proteins encoded by upregulated DEGs, blue boxes represent proteins from downregulated DEGs, and yellow boxes denote neighboring interacting proteins. The inset table lists the hub genes within our DEGs, ranked according to their scores from the degree algorithm.

Supplementary Table 1 Clinical and demographic characteristics of healthy donors (HD) and allergic patients (AP) in the replication cohort.

Supplementary Table 2 List of references and primers used for real time quantitative PCR.

Supplementary Table 3 DEGs in PBLs from *Olea europaea*-AP after *in vitro* challenge with 10 µg/ml T_9_ allergen. Genes were sorted using a threshold of an adjusted *p* value <0.1 and a log_2_FC=1 (FC = 2) for upregulated genes (shown above the dotted line) and log_2_FC= -1 (FC = 0.5) for downregulated genes (shown below the dotted line). Indicated by asterisk genes shared with T_9_-challenged healthy donors.

Supplementary Table 4 DEGs in PBLs from HD after *in vitro* challenge with 10 µg/ml T_9_ allergen. Genes were sorted using a threshold of an adjusted *p* value <0.1 and a log_2_FC=1 (FC = 2) for upregulated genes (shown above the dotted line) and log_2_FC= -1 (FC = 0.5) for downregulated genes (shown below the dotted line). Indicated by asteriks genes shared with T_9_-challenged allergic patients.
