# Supplementary material for: Elucidating molecular mechanisms of allergic sensitization to olive pollen through transcriptomics
Source: Front Immunol. 2026 Sep 4;17:1735045. doi: 10.3389/fimmu.2026.1735045 (PMC13585923; doi:10.3389/fimmu.2026.1735045)
Supplement: Supplementary file 1 [file Table1.docx]

**Supplementary Table 1. Clinical and demographic characteristics of healthy donors (HD) and allergic patients (AP) in the replication cohort.**

|  | **HD** | **AP** |
| --- | --- | --- |
| n | 5 | 5 |
| Age (max/min)* | 45.40 ± 6.08 (63-24) | 28.00 ± 4.18 (41-18) |
| Sex (male/female) | (4/1) | (2/3) |
| European ancestry | 100% | 100% |
| Total IgE (IU/ml)* | 41.26 ± 19.15 | 529.88 ± 300.84 |
| Positive skin tests | 0% | 100% |
| Olive-tree specific IgE (IU/ml)* | 0.00 ± 0.00 | 55.90 ± 18.23 |
| Ole e 1 specific IgE (IU/ml)* | 0.00 ± 0.00 | 42.48 ± 23.57 |
| Ole e 7 specific IgE (IU/ml)* | 0.00 ± 0.00 | 35.17 ± 16.94 |
| Ole e 9 specific IgE (IU/ml)* | 0.00 ± 0.00 | 11.04 ± 9.83 |

*Mean ± SEM

**Supplementary Table 2. List of references and primers used for real time quantitative PCR.**

| **Gene** | **Reference** | **Product size** |
| --- | --- | --- |
| *Mlxipl* | Hs00975714_m1 | 56 bp |
| *Shroom2* | Hs01113636_m1 | 61 bp |
| *Kcnma1* | Hs01119504_m1 | 75 bp |
| *Il31ra* | Hs00371172_m1 | 78 bp |
| *Areg* | Hs00950669_m1 | 66 bp |
| *Il18r1* | Hs00977691_m1 | 133 bp |
| *Tgm2* | Hs01096681_m1 | 129 bp |
| *Stc1* | Hs00174970_m1 | 81 bp |
| *Ccl1* | Hs00171072_m1 | 67 bp |
| *Gckr* | Hs00386984_m1 | 58 bp |
| *Grem1* | Hs00171951_m1 | 80 bp |
| *Clec4f* | Hs00403246_m1 | 68 bp |
| *rRNA18S* | Hs03003631_g1 | 69 bp |

**Supplementary Table 3. DEGs in PBLs from *Olea europaea*-AP after *in vitro* challenge with 10 µg/ml T_9_ allergen.** Genes were sorted using a threshold of an adjusted *p* value <0.1 and a log_2_FC=1 (FC=2) for upregulated genes (shown above the dotted line) and log_2_FC= -1 (FC= 0.5) for downregulated genes (shown below the dotted line). Indicated by asterisk genes shared with T_9_-challenged healthy donors.

| **Gene name** | **Gene symbol** | **fold change** | **adjusted *p*-value** |
| --- | --- | --- | --- |
| Dishevelled Associated Activator Of Morphogenesis 2* | *DAAM2* | 16.740 | 7.34E-08 |
| V-Set And Immunoglobulin Domain Containing 4* | *VSIG4* | 3.401 | 2.79E-05 |
| MLX interacting protein-like | MLXIPL | 3.311 | 7.20E-04 |
| T-box 3 | *TBX3* | 3.068 | 6.25E-03 |
| Epithelial Membrane Protein 2* | *EMP2* | 2.981 | 4.27E-04 |
| shroom family member 2 | *SHROOM2* | 2.942 | 8.36E-03 |
| dehydrogenase/reductase (SDR family) member 3 | *DHRS3* | 2.795 | 1.71E-08 |
| FKBP Prolyl Isomerase 5* | *FKBP5* | 2.779 | 6.97E-03 |
| prostaglandin E synthase | *PTGES* | 2.730 | 1.51E-06 |
| serine dehydratase | *SDS* | 2.704 | 2.79E-05 |
| uncharacterized PTPRN2-AS1 (PTPRN2-AS1), non-coding RNA* | *LOC100506585* | 2.690 | 4.70E-11 |
| C-type lectin domain family 10, member A | *CLEC10A* | 2.644 | 2.96E-03 |
| potassium channel, calcium activated large conductance subfamily M alpha, member 1 | *KCNMA1* | 2.589 | 6.89E-04 |
| interleukin 31 receptor A | *IL31RA* | 2.381 | 7.46E-04 |
| Tyrosylprotein Sulfotransferase 1* | *TPST1* | 2.364 | 7.65E-04 |
| Endothelin Receptor Type B* | *EDNRB* | 2.315 | 4.94E-04 |
| Potassium Voltage-Gated Channel Subfamily E Regulatory Subunit 1* | *KCNE1* | 2.287 | 6.49E-03 |
| Acetoacetyl-CoA Synthetase Pseudogene 1 | *AACSP1* | 2.281 | 2.23E-02 |
| Killer Cell Immunoglobulin Like Receptor, Three Ig Domains X1 (Pseudogene) | *KIR3DX1* | 2.195 | 4.49E-05 |
| Long Intergenic Non-Protein Coding RNA 2289 | *LINC02289* | 2.191 | 2.84E-03 |
| amphiregulin | *AREG* | 2.189 | 2.23E-02 |
| sialic acid binding Ig-like lectin 11 | *SIGLEC11* | 2.174 | 3.51E-04 |
| RNF103-CHMP3 readthrough | *RNF103-CHMP3* | 2.161 | 6.87E-02 |
| collagen, type IX, alpha 2 | *COL9A2* | 2.139 | 1.76E-02 |
| MROH7-TTC4 Readthrough (NMD Candidate) | *MROH7-TTC4* | 2.115 | 6.08E-02 |
| interleukin 18 receptor 1 | *IL18R1* | 2.105 | 3.80E-02 |
| olfactomedin-like 2B | *OLFML2B* | 2.092 | 1.08E-03 |
| StAR-related lipid transfer (START) domain containing 13 | *STARD13* | 2.083 | 6.61E-04 |
| transglutaminase 2 | *TGM2* | 2.083 | 1.16E-05 |
| progressive rod-cone degeneration | *PRCD* | 2.081 | 1.66E-02 |
| complement component 1, q subcomponent, C chain | *C1QC* | 2.066 | 8.88E-03 |
| calcium/calmodulin-dependent protein kinase kinase 1, alpha | *CAMKK1* | 2.055 | 1.26E-05 |
| hypermethylated in cancer 1 | *HIC1* | 2.053 | 2.41E-06 |
| potassium channel, inwardly rectifying subfamily J, member 10 | *KCNJ10* | 2.024 | 2.21E-02 |
| Small Integral Membrane Protein 10 Like 2A | *SMIM10L2A* | 0.498 | 2.18E-03 |
| Tripartite Motif-Containing 51E, Pseudogene* | *TRIM51EP* | 0.483 | 1.33E-02 |
| wingless-type MMTV integration site family, member 3 | *WNT3* | 0.483 | 5.04E-03 |
| phospholipase A2 inhibitor and LY6/PLAUR domain containing | *PINLYP* | 0.481 | 4.14E-04 |
| C-type lectin domain family 4, member F | *CLEC4F* | 0.445 | 4.98E-02 |
| gremlin 1, DAN family BMP antagonist | *GREM1* | 0.442 | 1.32E-02 |
| glucokinase (hexokinase 4) regulator | *GCKR* | 0.442 | 6.56E-03 |
| chemokine (C-C motif) ligand 1 | *CCL1* | 0.377 | 1.78E-02 |
| stanniocalcin 1 | *STC1* | 0.338 | 1.39E-02 |
| collagen, type VI, alpha 5 | *COL6A5* | 0.310 | 7.02E-03 |
| histone cluster 2, H4b | *HIST2H4B* | 0.087 | 1.61E-02 |
| PIGY upstream reading frame | *PYURF* | 0.003 | 3.66E-02 |

**Supplementary Table 4. DEGs in PBLs from HD after *in vitro* challenge with 10 µg/ml T_9_ allergen.** Genes were sorted using a threshold of an adjusted *p* value <0.1 and a log_2_FC=1 (FC=2) for upregulated genes (shown above the dotted line) and log_2_FC= -1 (FC= 0.5) for downregulated genes (shown below the dotted line). Indicated by asteriks genes shared with T_9_-challenged allergic patients.

| **Gene name** | **Gene symbol** | **fold change** | **adjusted *p*-value** |
| --- | --- | --- | --- |
| Salt Inducible Kinase 1B (Putative) | *SIK1B* | 2052.704 | 7.85E-03 |
| Survival Of Motor Neuron 2, Centromeric | *SMN2* | 373.285 | 5.83E-02 |
| Dishevelled Associated Activator Of Morphogenesis 2* | *DAAM2* | 8.291 | 6.26E-04 |
| V-Set And Immunoglobulin Domain Containing 4* | *VSIG4* | 3.054 | 1.28E-05 |
| Transmembrane And Immunoglobulin Domain Containing 3 | *TMIGD3* | 2.490 | 7.99E-03 |
| Epithelial Membrane Protein 2* | *EMP2* | 2.390 | 1.50E-02 |
| Solute Carrier Family 5 Member 9 | *SLC5A9* | 2.229 | 1.53E-02 |
| Macrophage Scavenger Receptor 1 | *MSR1* | 2.138 | 1.15E-03 |
| Potassium Voltage-Gated Channel Subfamily E Regulatory Subunit 1* | *KCNE1* | 2.092 | 1.58E-03 |
| FKBP Prolyl Isomerase 5* | *FKBP5* | 2.069 | 2.06E-02 |
| Endothelin Receptor Type B* | *EDNRB* | 2.053 | 5.87E-03 |
| uncharacterized PTPRN2-AS1 (PTPRN2-AS1), non-coding RNA* | *LOC100506585* | 2.046 | 5.93E-05 |
| Tyrosylprotein Sulfotransferase 1* | *TPST1* | 2.044 | 3.32E-03 |
| Tripartite Motif-Containing 51E, Pseudogene* | *TRIM51EP* | 0.460 | 8.36E-03 |
| BCL2L2-PABPN1 Readthrough | *BCL2L2-PABPN1* | 0.001 | 1.32E-02 |
